# Microglia contribute to the production of the amyloidogenic ABri peptide in familial British dementia

**DOI:** 10.1007/s00401-024-02820-z

**Published:** 2024-11-15

**Authors:** Charles Arber, Jackie M. Casey, Samuel Crawford, Naiomi Rambarack, Umran Yaman, Sarah Wiethoff, Emma Augustin, Thomas M. Piers, Matthew Price, Agueda Rostagno, Jorge Ghiso, Patrick A. Lewis, Tamas Revesz, John Hardy, Jennifer M. Pocock, Henry Houlden, Jonathan M. Schott, Dervis A. Salih, Tammaryn Lashley, Selina Wray

**Affiliations:** 1 Department of Neurodegenerative Disease, UCL Queen Square Institute of Neurology, London, UK; 2 Dementia Research Institute at UCL, London, UK; 3 Klinik für Neurologie mit Institut für Translationale Neurologie Albert Schweitzer Campus, Gebäude A1, 48149 Münster, Germany; 4 Department of Neuroinflammation, UCL Queen Square Institute of Neurology, London, UK; 5 Department of Pathology, New York University Grossman School of Medicine, New York, USA; 6 Royal Veterinary College, Royal College Street, London, UK; 7 The Queen Square Brain Bank for Neurological Disorders, Department of Clinical and Movement Neuroscience, UCL Queen Square Institute of Neurology, London, UK; 8 Department of Neuromuscular Disorders, UCL Queen Square Institute of Neurology, London, UK; 9 Dementia Research Centre, UCL Queen Square Institute of Neurology, London, UK

**Keywords:** Amyloid, Dementia, iPSC, Microglia, Alzheimer’s disease, Familial British dementia

## Abstract

Mutations in *ITM2B* cause familial British, Danish, Chinese, and Korean dementias. In familial British dementia (FBD), a mutation in the stop codon of the *ITM2B* gene (also known as *BRI2*) causes a C-terminal cleavage fragment of the ITM2B/BRI2 protein to be extended by 11 amino acids. This fragment, termed amyloid-Bri (ABri), is highly insoluble and forms extracellular plaques in the brain. ABri plaques are accompanied by tau pathology, neuronal cell death and progressive dementia, with striking parallels to the aetiology and pathogenesis of Alzheimer’s disease. The molecular mechanisms underpinning FBD are ill-defined. Using patient-derived induced pluripotent stem cells, we show that expression of *ITM2B/BRI2* is 34-fold higher in microglia than neurons and 15-fold higher in microglia compared with astrocytes. This cell-specific enrichment is supported by expression data from both mouse and human brain tissue. ITM2B/BRI2 protein levels are higher in iPSC-microglia compared with neurons and astrocytes. The ABri peptide was detected in patient iPSC-derived microglial lysates and conditioned media but was undetectable in patient-derived neurons and control microglia. The pathological examination of post-mortem tissue supports the presence of ABri in microglia that are in proximity to pre-amyloid deposits. Finally, gene co-expression analysis supports a role for ITM2B/BRI2 in disease-associated microglial responses. These data demonstrate that microglia are major contributors to the production of amyloid forming peptides in FBD, potentially acting as instigators of neurodegeneration. Additionally, these data also suggest ITM2B/BRI2 may be part of a microglial response to disease, motivating further investigations of its role in microglial activation. These data have implications for our understanding of the role of microglia and the innate immune response in the pathogenesis of FBD and other neurodegenerative dementias including Alzheimer’s disease.

**Supplementary Information:**

The online version contains supplementary material available at 10.1007/s00401-024-02820-z.

## Introduction

Mutations in *ITM2B* (also known as *BRI2*) cause familial British [68], Danish [69], Chinese [35] and Korean [52] dementias (FBD, FDD, FCD, and FKD, respectively) and have also been associated with autosomal dominant retinal dystrophy [5]. ITM2B/BRI2 is a type II transmembrane protein that is cleaved by FURIN convertase to release an extracellular 23 amino acid C terminal fragment [29, 59]. Dominantly inherited mutations linked to dementia increase the length of this C terminal fragment to 34 amino acids, either by disrupting the stop codon (in FBD, FCD and FKD), or due to a 10 nucleotide duplication immediately upstream of the stop codon (in FDD). These extended peptides are amyloidogenic and termed Amyloid-Bri (ABri) in FBD and Amyloid-Dan (ADan) in FDD; no histopathological data are available for FCD or FKD to date, but the mutations are predicted to produce a peptide that differs from ABri by a single amino acid. These amyloidogenic peptides aggregate to form extracellular amyloid deposits [13] and are thus theorised to cause neurodegeneration in a manner analogous to amyloid-beta (Aβ) in Alzheimer’s disease (AD) [7, 38].

The common clinical features of FBD and FDD include progressive dementia and cerebellar ataxia [73]. Additional specific features include spastic paraparesis in FBD, as well as cataracts and hearing defects in FDD [35, 47, 63]. Pathologically, FBD and FDD are characterised by extensive amyloid angiopathy, amyloid plaques, and pre-amyloid deposits in multiple brain regions. ABri and ADan also accumulate in organs throughout the body [14]. Similar to AD, and supporting shared aetiology, neurofibrillary tangle tau pathology is common in both FBD and FDD [24, 25].

The normal function of ITM2B/BRI2 is not well understood. The protein is localised to the plasma membrane and potentially the mitochondrial inner membrane [72] and has been reported to interact with APP and Aβ—modulating Aβ deposition [10, 28, 64]. The conserved BRICHOS domain, which is distinct from the cleaved C-terminus fragment, is able to reduce the fibrillation of amyloids [46, 71]. Conversely, APP was shown to be a molecular effector of ADan-associated synaptic and memory deficits, as APP haploinsufficiency prevents synaptic and memory deficits in a mouse model of FDD [64]. Aβ co-accumulates with ADan pathology but this has not been observed with ABri [25, 40, 66]. Mechanistically, there remains uncertainty whether mutations cause a loss of normal function of the ITM2B/BRI2 protein, as knock-in mouse models have shown reduced expression of ITM2B/BRI2 protein [64, 75].

Genome-wide association studies have revealed the importance of microglia in the determination of risk for several neurodegenerative disorders, particularly Alzheimer’s disease [48, 70]. Further, heterozygous mutations in *TREM2*, a microglial gene, increase risk for AD three-fold [16, 26]. The molecular mechanisms by which microglia contribute to dementia onset and progression is an area of intense investigation. Recent work suggests that microglia regulate the transition of amyloid pathology to tau pathology [32, 33] via genes expressed by the amyloid-responsive microglial state (ARM) or the disease-associated microglial state (DAM)[27, 37, 41, 54]; cell states that are enriched in dementia and driven by genes including *APOE* and *TREM2*.

In this study, we sought to gain insights into the cellular consequences of FBD-associated *ITM2B/BRI2* mutations by developing patient-derived iPSC models of FBD. Based on previous pathological findings that ITM2B/BRI2 can be expressed by neurons and glia [31], we sought to determine the effect of FBD mutations on ITM2B/BRI2 in different cell types. Due to the parallels between FBD, FDD and AD, and the crucial role of microglia in AD progression [19, 23, 48], we then investigated a possible role of microglia in FBD and FDD pathobiology.

## Materials and methods

### Cell Culture

All components were ThermoFisher, unless stated. All growth factors were Peprotech, unless stated. Patient-derived fibroblasts were obtained from a skin biopsy with ethical approval from the Institute of Neurology joint research ethics committee at the Hospital for Neurology and Neurosurgery (10/H0721/87) with informed consent (Table 1). Fibroblasts were grown in DMEM supplemented with 10% FBS and passaged using 0.05% trypsin. Fibroblasts, below passage 4, were reprogrammed using episomal reprogramming as described previously [43]. Episomal plasmids, obtained from Addgene #27,077, #27,078 and #27,080, were electroporated into fibroblasts using Lonza P2 Nucleofection. 7 days post electroporation, media was changed to Essential 8 and iPSC colonies appeared after a subsequent 20 days. iPSC colonies were picked manually and expanded in Essential 8 media, on geltrex substrate and passaged manually using 0.5 mM EDTA. Ctrl1 and Ctrl2 refer to the well characterised RBi001-a and SIGi1001-a-1, respectively, both available via Sigma Aldrich [3, 4].

**Table 1:** Details of stem cell lines

	Gender	Age at biopsy	FBD status	Source
FBD fibroblast donor 1 (FBD1)	Female	50	FBD -affected	UCL DRC
FBD fibroblast donor 2 (FBD2)	Male	58	FBD -affected	NHNN
Ctrl1 (RBi001-a)	Male	45–49	Unaffected	Sigma Aldrich/EBiSC
Ctrl2 (SIGi1001-a-1)	Female	20–24	Unaffected	Sigma Aldrich/EBiSC
hESC (Shef6)	Female	0	Unaffected	UK stem cell bank

*FBD* familial British dementia, *DRC* dementia research centre, *NHNN* national hospital for neurology and neurosurgery

Genomic DNA was isolated from iPSC clones using cell lysis buffer containing 0.5% SDS and 0.5 mg/ml proteinase k. Samples were lysed at 55 °C overnight and DNA was extracted using phenol–chloroform extraction with ethanol-based precipitation. DNA was quantified using nanodrop, diluted to 50 ng/µl in 15 µl and put forward to Sanger sequencing using standard PCR master mix in a touchdown-PCR.

Karyotype stability was confirmed by The Doctors Laboratory (London, UK) using G-band analysis. Additionally, low coverage whole genome sequencing was performed, where the genome was divided into 1000 kb bins and reads were mapped within each bin. The QDNASeq package was used [56] and sequencing was performed with UCL Genomics. A stem cell phenotype was confirmed via comparing the expression of 770 genes associated with pluripotency and differentiation with a panel of 3 established iPSC/hESC (Ctrl1, Ctrl2 and Shef6 [4]) lines using the Nanostring Stem Cell Characterisation Panel.

iPSCs were differentiated to cortical neurons using established protocols [2, 4, 61]. Briefly, iPSCs at 100% confluence were subject to neural induction using dual SMAD inhibition in N2B27 media (1 μM dorsomorphin and 10 μM SB431542, both TOCRIS). N2B27 media consists of 50% DMEM-F12, 50% Neurobasal supplemented with 0.5X N2 supplement, 0.5X B27 supplement, 0.5X L-glutamine, 0.5X non-essential amino acids, 0.5X penicillin/streptomycin, insulin (25U) and β-mercaptoethanol (1:1000). Cultures were passaged using Dispase at 10DIV and again at 18DIV. Progenitors underwent a final passage using Accutase at 35DIV and neuronal maturation was performed in N2B27 media. Day 90 was taken as the final time point.

iPSCs were differentiated to astrocytes following established protocols [18]. Neuronal cultures (90DIV iPSC-neuronal cultures generated as above) were enriched for astrocytes via continuous EDTA passaging in N2B27 media containing 10 ng/ml FGF2 (Peprotech). The cells underwent a gliogenic switch at around 110DIV. At 150 days in vitro, a final 2-week maturation step involved BMP4 (10 ng/ml) and LIF (10 ng/ml) in N2B27 media.

iPSCs were differentiated to microglia following established methods [12, 74]. Briefly, myeloid embryoid bodies (EBs) were produced using 10,000 cells in Essential 8 media supplemented with 50 ng/ml BMP4, 50 ng/ml VEGF and 20 ng/ml SCF. After 4 days myeloid EBs were maintained in X-Vivo 15 media supplemented with 100 ng/ml MCSF and 25 ng/ml IL3. After 4 weeks, microglia-like cells were released from the EBs into the media. Microglia-like cells were harvested weekly and matured using DMEM-F12 media supplemented with 100 ng/ml IL34, 25 ng/ml MCSF and 5 ng/ml TGFβ1. A final maturation step was performed via a 2-day treatment with CX3CL1 (100 ng/ml) and CD200 (100 ng/ml). For cell treatments, IFNβ (10 ng/ml) or TNFα (10 ng/ml) was added to fresh media for 24 h versus vehicle control (PBS), and LPS (100 ng/ml, Sigma) was added for 6 h versus vehicle control.

### qPCR

RNA was extracted from cells using Trizol, following the manufacturer’s protocol. 2 µg of RNA was reverse transcribed using SuperScript IV reverse transcriptase, random hexamer mix and RNAse OUT. qPCR was run on an Agilent Aria MX using POWER Sybr green master mix. The primers are shown in Table 2.

**Table 2:** Primers used in this study

Target	Forward	Reverse	Amplicon
*ITM2B*	CGTGAAGCCAGCAATTGTTTCGCA	AGCCCTGTTTGCTACTTACATG	191 bp
*FURIN*	GATCGTGACGACTGACTT	TATGAGTGGCTCACTTTCC	222 bp
*RPL18a*	CCCACAACATGTACCGGGAA	TCTTGGAGTCGTGGAACTGC	180 bp
*OCT4*	TTCTGGCGCCGGTTACAGAACCA	GACAACAATGAAAATCTTCAGGAGA	218 bp
*SOX2*	CATGGCAATCAAAATGTCCA	TTTCACGTTTGCAACTGTCC	119 bp
*NANOG*	GCTTGCCTTGCTTTGAAGCA	TTCTTGACTGGGACCTTGTC	256 bp
*DNMT3B*	TTTAGGGAGAACGGGAAT	AGCACCAGTAAGAAGAGT	88 bp
*S100A4*	TTCTTTCTTGGTTTGATCC	TTAGTTCTGACTTGTTGAGC	211 bp
*VIM*	GTACGTCAGCAATATGAAAG	AGTGTCTTGGTAGTTAGCAG	270 bp
*TREM2*	GGAGTCTGAGAGCTTCGAGGATG	TTCACTGGGTGGATGTGTCCC	196 bp
*CTSB*	ATACAATTCCTACAGCGTCT	GTGTTGGTACACTCCTGACT	130 bp
*P2YR12*	GGTCAGATTACAAGAGCAC	TGATAACTGTTGATTCTGGA	178 bp
*IL1B*	CTTCAGCCAATCTTCATT	CACTGTAATAAGCCATCAT	88 bp
*IL10*	GTGGAGCAGGTGAAGAAT	TCTATGTAGTTGATGAAGATGTC	92 bp

### Immunocytochemistry

Cells were fixed in 4% paraformaldehyde for 15 min. Cells were then washed thrice in 0.3% Triton-X-100 in PBS (PBSTx) prior to blocking in 3% bovine serum albumin in PBSTx. Primary antibodies (Table 3) were incubated in blocking solution overnight. After three subsequent washes in PBSTx, secondary antibodies (AlexaFluor) were incubated for 1 h in the dark in blocking solution. After a final three washes and exposure to DAPI nuclear stain, images were taken on a Zeiss LSM microscope. No post-hoc image adjustments were made.

**Table 3:** Antibodies used in this study

Target	Species	Company	RRID
SSEA4	mouse	BioLegend 330,401	AB_1089209
OCT4	goat	Santa Cruz sc-5297	AB_628051
TBR1	rabbit	Abcam ab31940	AB_2200219
TUJ1	mouse	BioLegend 801,201	AB_2313773
IBA1	goat	Abcam ab107159	AB_10972670
ITM2B/BRI2 CTF	rabbit	Sigma HPA029292	AB_10601917
ITM2B/BRI2 NTF	mouse	Santa cruz sc-374362	AB_10988049
TREM2	rabbit	Cell Signaling Tech 91,068	AB_2721119
SOX9	Rabbit	Abcam ab185966	AB_2728660
Actin	mouse	Sigma A1978	AB_476692
GAPDH	mouse	Ambion AM4300	AB_437392
ABri	rabbit	338[68]	
ADan	rabbit	5282[69]	
CD68	mouse	Dako M0876	AB_2074844
CR3/43	mouse	Dako M0775	AB_2313661

### Immunohistochemistry

Details of tissue donors provided in Table 4. Formalin fixed paraffin embedded sections were deparaffinized in xylene, followed by rehydration using graded alcohols (100%, 95% and 70%). For all immunohistochemical staining the endogenous peroxidase activity was blocked using 0.3% H_2_O_2_ in methanol for 10 min. Sections were subjected to various pre-treatments, depending on the antibody used. For ABri and ADan, sections were pre-treated with formic acid for 10 min. For microglial staining (CD68 or CR3/43), sections were pressure cooked in citrate buffer pH6.0 for 10 min. Non-specific protein binding was blocked using 10% non-fat milk in PBS (0.05 M pH7.2) by incubating the sections for 30 min at room temperature. Sections were incubated with the required primary antibody (ABri: 338 1:1000 from Ghiso lab; ADan: 5282 1:1000 from Ghiso lab; CD68: 1:150 DAKO; CR3/43: 1:100 DAKO) for 1 h at room temperature. Incubation with the relevant biotinylated secondary antibody (Vector) was carried out for 30 min at room temperature. Sections were incubated in avidin–biotin complex (ABC, Dako) for 30 min and the antigen–antibody reaction was visualized using di-aminobenzidine (DAB, Sigma) as the chromogen. Sections were counterstained with Mayers haematoxylin (BDH), dehydrated and mounted. Tissue sections were digitally scanned using an Olympus VS120 slide scanner at 20 × magnification.

**Table 4:** Details of tissue donors

Case	Gender	AAO	AAD	Disease duration	Neuro-pathological diagnosis	Aβ + / −	Braak and Braak tau Stage	CAA ±	Reference
1	F	–	86	–	Control	−	–	−	–
2	F	57	68	11	FBD	−	V	+	Case 5 – [24]
3	M	40	58	18	FDD	+	V-VI	+	Case 3 – [25]

Aβ and CAA staging is characterised as present or absent as the pathology does not follow staging criteria for Alzheimer’s disease

*FBD* familial British dementia, *FDD* familial Danish dementia, *QSBB* queen square brain bank for neurological disorders

### In situ hybridisation

Flash-frozen tissue sections from the frontal cortex of a FBD case were cut at 15 μm. For the in-situ hybridization experiment, a DIG-labelled oligonucleotide probe corresponding to the region 28–68 of the BRI2 gene was used. The probe was labelled using the DIG Oligonucleotide 3’-end labelling kit (Roche), 100 pmol of the oligonucleotide was labelled according to the manufacturer’s instructions. The tissue sections were fixed in 4% paraformaldehyde in PBS for 5 min, washed, dehydrated, and hybridized with the DIG-labelled probe overnight at 37 °C in a humidified chamber. Post-hybridization washes were performed using 1 × SSC solution at 55 °C and room temperature to remove unbound probe and salts. For visualization, sections underwent immunohistochemistry using an anti-DIG antibody (Roche, 1:250 dilution), followed by incubation with biotinylated anti-mouse antibody (Dako, 1:200 dilution) and ABC reagent. Visualization was carried out using the Glucose Oxidase Nickel DAB method. Finally, sections were counterstained with 0.1% Nuclear Fast Red, dehydrated, cleared, and mounted. Control experiments included the use of antisense probes, competition assays with excess unlabelled probe, and replacement of primary and secondary antibodies with PBS to ensure specificity of staining. All controls yielded negative or significantly diminished staining, validating the specificity of the in-situ hybridization and immunohistochemistry methods.

### Thioflavin staining

Thioflavin staining was used to demonstrate protein deposits in amyloid conformation and used in addition to immunohistochemical staining. Once immunohistochemical staining was complete sections were incubated with thioflavin solution (0.1% aqueous solution) for 7 min and differentiated with 70% alcohol, followed by washing in distilled water.

### Western blotting

Cells were lysed in RIPA buffer with protease and phosphatase inhibitors (Roche) and centrifuged to remove insoluble debris. Protein content was quantified via BCA assay (BioRad). Samples were denatured at 95 °C for 5 min in LDS buffer with DTT and loaded on 4–12% precast polyacrylamide gels. Gels were transferred to nitrocellulose membranes and blocked, using PBS with 0.1% Tween-20 (PBSTw) and 3% BSA. Primary antibodies were incubated in blocking solution overnight, washed thrice in PBSTw and incubated with secondary antibodies for 1 h. After three final washes, images were captured on a LiCor Odyssey fluorescent imager.

### Gene coexpression analysis

We employed online databases on human tissue including HumanBase (human macrophages, top 20 genes, minimum interaction prediction confidence: 0.65), GeneFriends (human brain tissue, top 10 genes, Pearson correlation threshold: 0.85) and COXPRESdb v8 (non-specific tissue, top 10 co-expressed genes ranked based on z-scores) to generate co-expression networks for *ITM2B* (Table 5). These lists were analysed to determine the genes which were present in multiple databases.

**Table 5:** Online platforms for co-expression network analysis

Platform		Description
HumanBase 1.0 (formerly GIANT) [15]	https://humanbase.flatironinstitute.org/gene/	A resource that includes genome-scale functional maps and tissue-specific networks across 144 human tissues with statistical prioritisation developed using Bayesian framework on more than 14,000 publications. Human macrophage tissue was selected for this analysis
GeneFriends [50]	https://www.genefriends.org/	A guilt-by-association data-driven analysis platform using RNAseq based gene co-expression network construction with functional annotation for multiple species. Human brain tissue was selected for this analysis
COXPRESdb v8 [42]	https://coxpresdb.jp/	A gene coexpression database based on DNA microarray analysis and over 200,000 RNASeq runs which collates data to present coexpression relationships based on relative expression patterns of genes and networks and protein–protein-interactions. This platform also uses the KEGG pathway and Gene Ontology Biological Process scores to substantiate the results

The ITM2B gene network (Fig. 4) was generated through a co-expression analysis of pre-processed Pearson’s residuals obtained from a microglial single cell RNA-seq dataset collected from the dorsolateral prefrontal cortex of AD and mild cognitively impaired (MCI) brains [44]. The dataset was filtered to remove cells that appeared unhealthy or potential doublets, with cells having greater than 5% ratio of mitochondrial to total counts or less than 1000 counts or less than 700 genes detected being removed using Seurat’s subset function. Additionally, samples from non-demented individuals with epilepsy were removed from the analysis, as they are not fully representative of healthy control brain. Genes showing low variation in expression between cells (coefficient of variation for Pearson’s residuals < 15%) were also removed, as they are not informative for co-expression analysis. The co-expression analysis was performed using the “getDownstreamNetwork” function from CoExpNets, which is an optimized version of the popular weighted gene co-expression network analysis (WGCNA) package [6]. This optimization involves a k-means clustering step to re-categorize genes into biologically relevant and reproducible modules. The mean log2 normalized expression of the most central genes within the “turquoise” module was used to determine *ITM2B* as one of the hub genes in the network. These genes were ranked based on their module membership (MM) scores, which were calculated using the “getMM” function from CoExpNets. The correlation matrix of the expression data was then used to rank the most connected genes to *ITM2B* within the module. The resulting network was visualized using the Cytoscape v.3.9.1 software.

### Gene knockdown

*ITM2B/BRI2* was knocked down in iPSC-derived microglial-like cells using DharmaFECT 1 (Horizon) using SMARTpool siRNA (Horizon) alongside scrambled, non-targeting siRNA. Briefly, cells were plated at 500,000 cells per well of a six-well plate. siRNA was prepared in DharmaFECT 1 (2.5 µl per well) in serum free media for 20 min, before evenly distributing onto cells. Protein lysates were taken 72 h later and run for Western blots, as above.

### Statistical analysis

Data were analysed in Microsoft Excel and Graphpad Prism. Statistical significance was calculated after normality testing using either two-way ANOVA tests with multiple comparisons testing or two tailed *t* tests, as described in figure legends.

## Results

### Patient-derived microglia generate the amyloidogenic ABri peptide

**Fig. 1:**
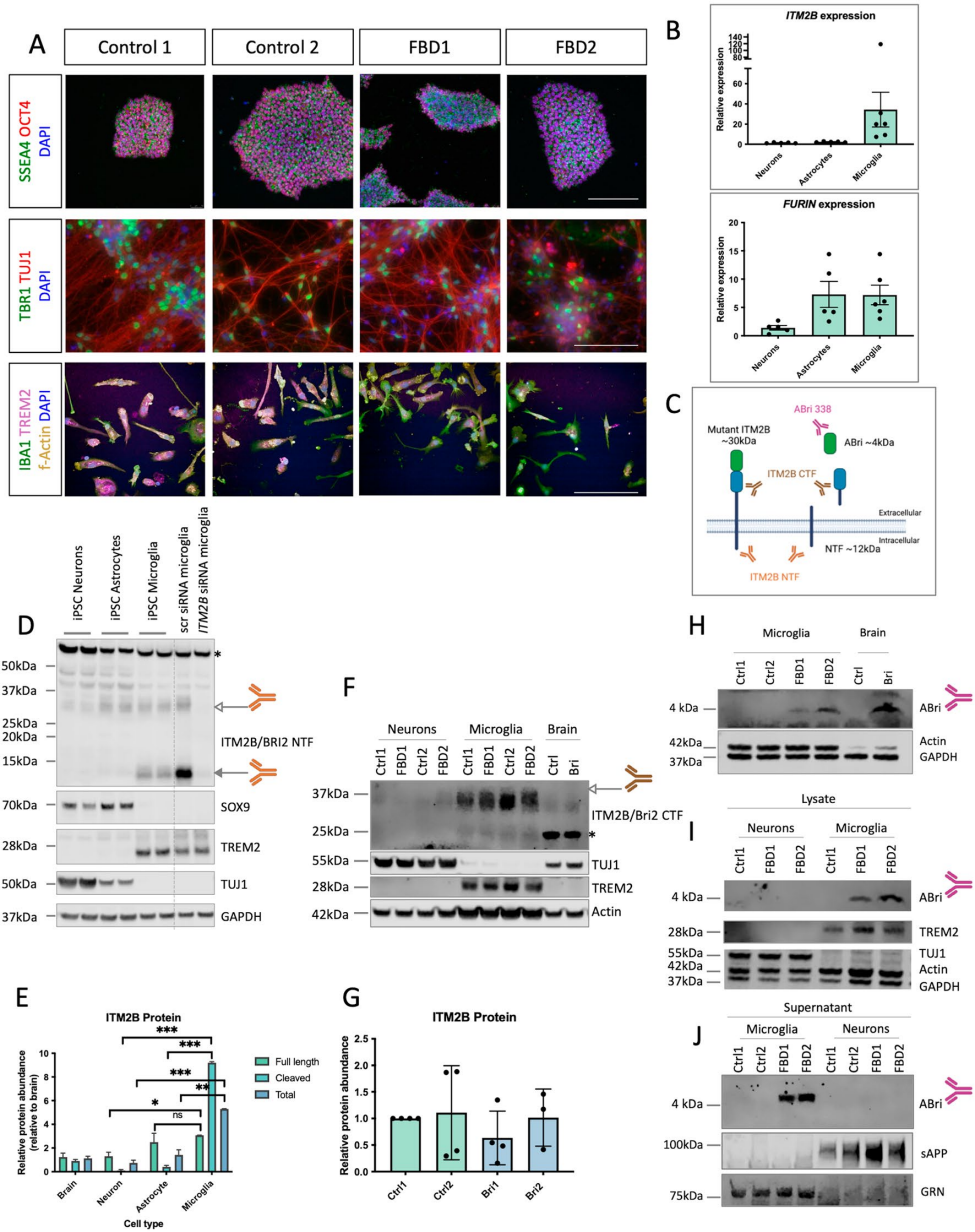
ABri is produced by iPSC-derived microglia. **A** Immunocytochemistry of iPSCs (upper panels), iPSC-derived neurons (middle panels) and iPSC-derived microglia (lower panels). SSEA4 and OCT4 are pluripotency markers, scale bar 200 μm. TUJ1 is a pan-neuronal marker and TBR1 labels deep layer cortical neurons, scale bar 200 μm. IBA1 labels microglia-like cells, scale bar 50 μm. **B** qPCR analysis of *ITM2B/BRI2* and *FURIN* expression in control iPSC-derived cortical neurons, astrocytes and microglia. Neuronal cDNA represents 5 independent inductions with 2 independent control iPSC lines, astrocytic cDNA was generated from 5 independent inductions of two independent control iPSC lines and microglial cDNA was generated from 6 harvests from 4 inductions and represents two independent control iPSC lines. Significant differences are abolished by the outlier at two standard deviations above the mean. **C** Representation of antibodies used in this figure (produced in biorender). **D** Western blotting of iPSC-derived neurons, iPSC-derived astrocytes and iPSC-derived microglia. *ITM2B/BRI2* knockdown via siRNA depicts antibody specificity for bands at around 30 kDa and 12 kDa. TUJ1, SOX9 and TREM2 are markers for neurons, astrocytes and microglia respectively. Samples represent two independent control lines for each cell type. Microglial samples from independent batches are separated by a dotted line. **E** Quantification of specific bands (30 kDa and 12 kDa) from 3 independent neuronal, astrocyte and microglia inductions of at least two control lines in each cell type. **F** Western blotting of iPSC neurons, iPSC microglia and post-mortem brain tissue for ITM2B/BRI2 as well as neuronal TUJ1, microglial TREM2 and loading control (Actin). **G** Quantification of Western blotting of ITM2B/BRI2 in control and patient-derived microglia from four harvests from three independent batches of microglia. **H**–**J**) Western blotting for ABri in iPSC-derived microglia lysates and brain tissue showed a band of 4 kDa. **J**) Western blotting for ABri in iPSC-derived microglial conditioned media, with secreted APP (sAPP) and GRN as neuronal and microglial loading controls respectively. White arrowheads show full length ITM2B/BRI2, grey arrowheads show cleaved fragments of ITM2B/BRI2 and asterisks show potential unspecific bands. Comparisons represent two tailed *t*-tests where * = *p* < 0.05, ** = *p* < 0.01, *** = *p* < 0.001

To generate a human, physiological model of FBD, we reprogrammed fibroblasts from two individuals with the FBD mutation, c.799 T > A (for donor details, see Table 1). iPSCs showed characteristic morphology and expression of the pluripotency markers SSEA4, OCT4, SOX2, NANOG and DNMT3B via immunocytochemistry and qPCR (Figs. 1A, S1A). Reprogrammed cells also showed appropriate downregulation of the fibroblast specific markers *S100A4* and *VIM* (Fig S1B). FBD iPSCs were further characterised to confirm the presence of the FBD mutation TGA > AGA (Fig S1C), as well as a stable karyotype (Fig S1D, E). Analysis of a panel of 770 genes associated with pluripotency and early differentiation demonstrated that the two FBD iPSC lines showed a global expression profile comparable to a panel of 3 control stem cell lines (Fig S1F).

We investigated the cell type specific expression of *ITM2B/BRI2* in control iPSC-derived neurons, astrocytes and microglia (Fig. 1A, B). Successful differentiation of iPSCs to cortical neurons was confirmed by morphology and expression of neuronal-specific TUJ1 and deep layer cortical neuronal marker TBR1. IBA1 expression confirmed the successful differentiation of iPSCs to microglia-like cells. Astrocyte differentiation was confirmed by the enrichment of SOX9 (Fig. 1D) and GFAP (Fig S2). qPCR analysis demonstrated that expression of *ITM2B/BRI2* was on average 34-fold higher in microglia when compared with neurons (Fig. 1B) and 14-fold higher in microglia compared with astrocytes. To determine if microglial enrichment of *ITM2B/BRI2* is due to insufficient maturation status of iPSC-derived cultures, we mined expression data from mouse and human brain tissue in seven freely available datasets and further confirmed a microglial enrichment of *ITM2B/BRI2* in the brain [11, 17, 22, 30, 36, 55, 77, 78] (Fig S3, S4). *FURIN*, encoding the enzyme responsible for cleavage of ITM2B/BRI2 in normal physiology and release of ABri and ADan in disease, showed enrichment in microglia and astrocytes relative to neurons (Fig. 1B). Using a panel of antibodies (Fig. 1C), western blotting of neuronal, astrocytic and microglial lysates reinforced the finding that ITM2B/BRI2 is highly expressed by microglia (Fig. 1D, E). The specificity of bands at around 30 kDa and 12 kDa, corresponding to full-length and cleaved ITM2B/BRI2 protein respectively, were confirmed by siRNA knockdown of *ITM2B/BRI2* in iPSC-derived microglia (Figs. 1D, S5). This finding is reinforced by a published proteomic study that shows relative depletion of ITM2B/BRI2 protein in acutely isolated mouse neurons compared with glia [60] (Fig S6). We also detected a band at around 25 kDa in human brain samples (Fig. 1F); this does not correspond to a known cleavage fragment of ITM2B/BRI2, and we cannot determine if this band is specific.

FBD and FDD mutations have been shown to reduce the levels of ITM2B/BRI2 protein in mouse knock-in models [64, 75]. To determine if the FBD mutation affects protein levels in iPSC-derived microglia, we performed Western blotting and observed comparable protein levels in patient-derived microglia and control microglia (Fig. 1F, G), albeit with a degree of variability between microglial batches.

Western blotting using the ABri-specific antibody (Ab338) was able to detect the presence of the 4 kDa ABri peptide in patient-derived microglial lysates (Fig. 1H, I) as well as in patient-derived microglial-conditioned media (Fig. 1J). We were not able to detect the ABri peptide in FBD neuronal lysates or control microglia (Fig. 1H–J).

### Post mortem immunohistochemistry shows colocalization of ABri and microglial markers

**Fig. 2:**
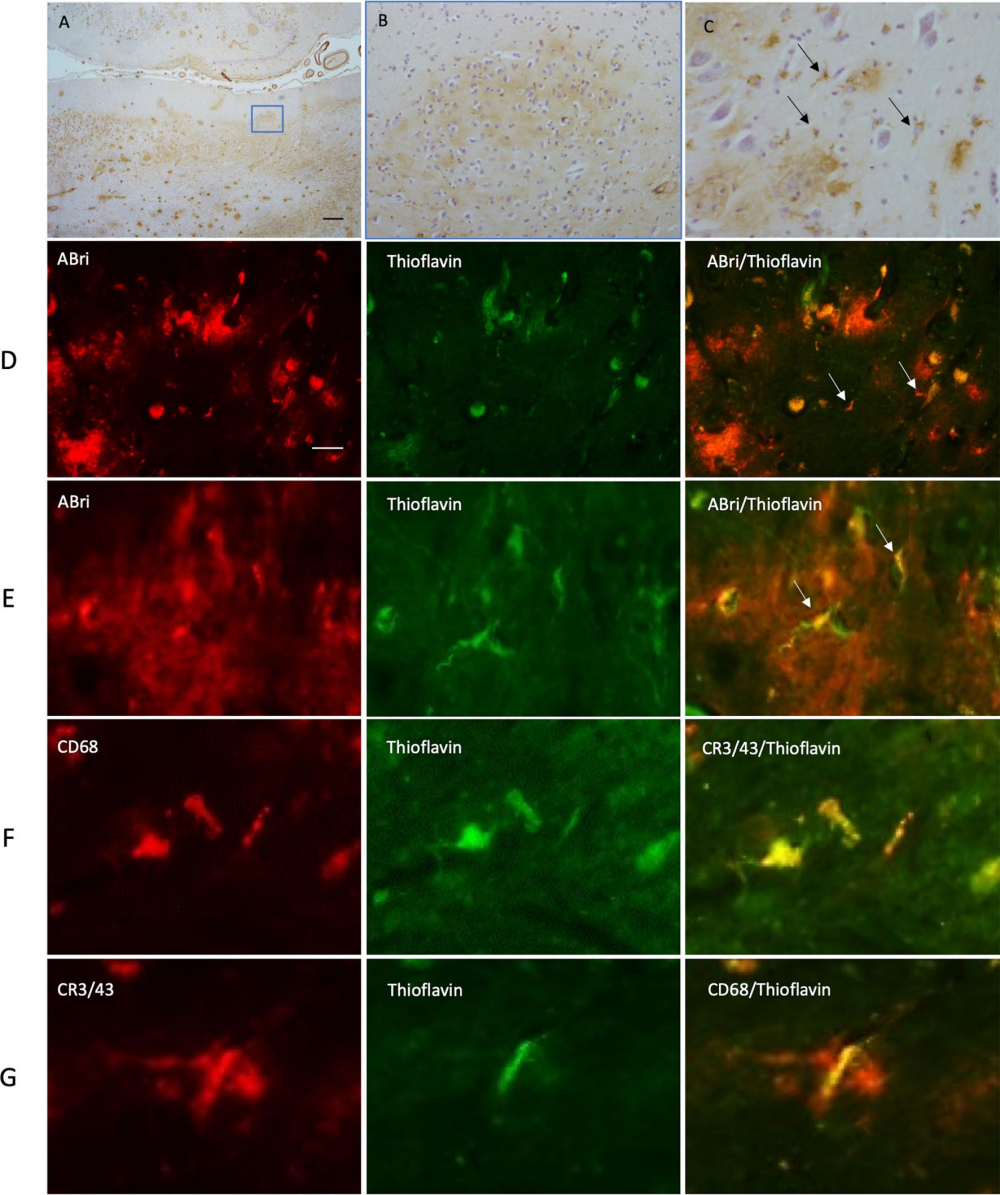
Immunohistochemical staining in FBD for ABri, Thioflavin and microglial markers. ABri pathology is observed in the hippocampus (**A**) in the form of extracellular amyloid and preamyloid deposits. ABri is also found within parenchymal and leptomeningeal blood vessels as cerebral amyloid angiopathy. ABri pre-amyloid plaques are shown at higher magnification in (**B**). The preamyloid deposits contained cells resembling microglia morphology (**C**, arrows). The bar represents 500 µm in (**A**) and 50 µm in (**B**) and 25 µm in (**C**). ABri immunohistochemistry (red, row **D** and **E**) combined with Thioflavin staining highlights the presence of ABri in cells resembling microglia. Microglial antibodies were used to determine the Thioflavin positive structures identified in the cells (rows **F** and **G**). The bar represents 100 µm in row (**D**) and 20 µm in row (**E**) and 10 µm rows in (**F**) and (**G**). Data are from one donor

**Fig. 3:**
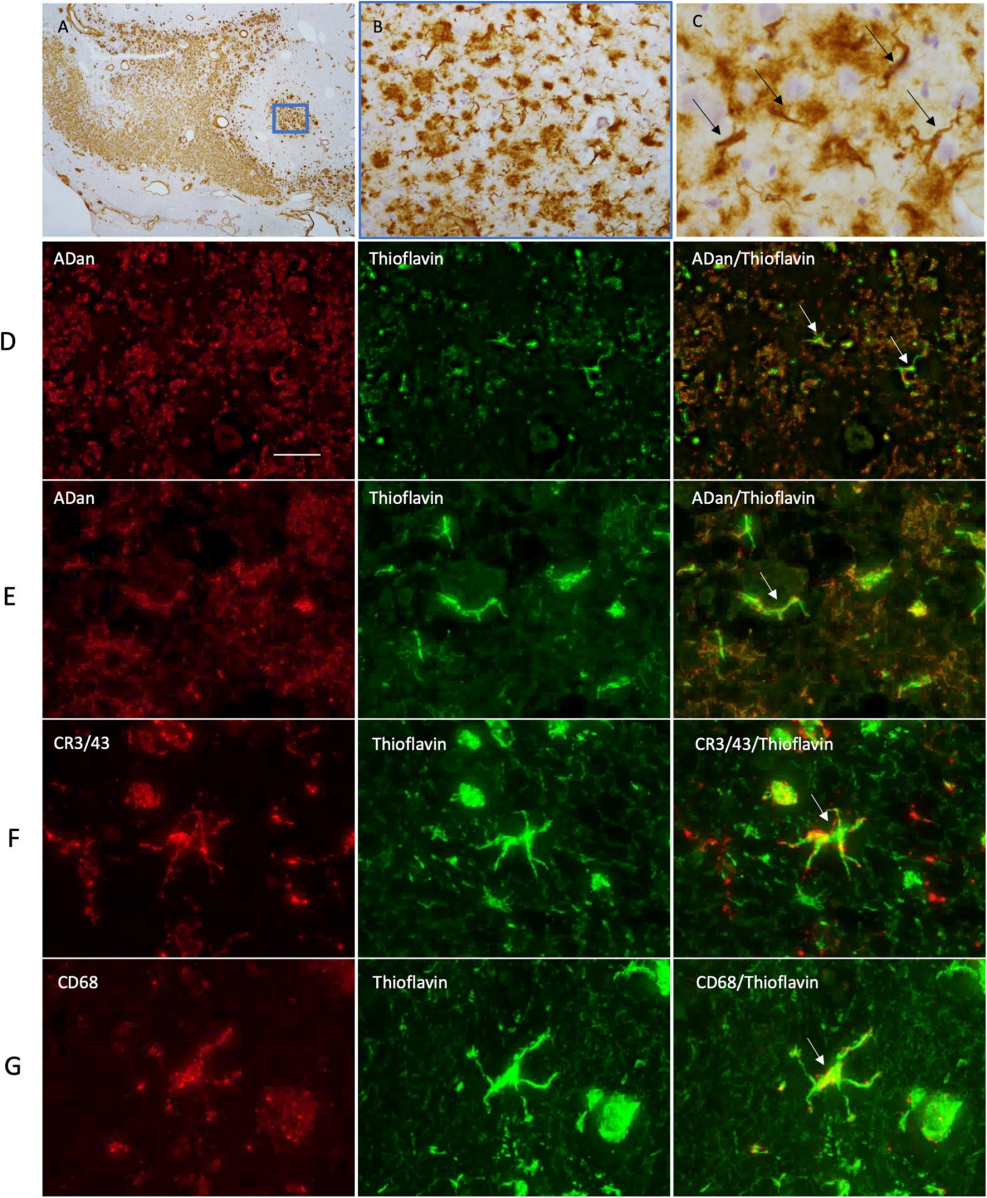
Immunohistochemical staining in FDD for ADan, thioflavin and microglial markers. ADan pathology was observed in the hippocampus (**A**) in the form of extracellular pre-amyloid deposits and cerebral amyloid angiopathy. At higher magnification we observed the preamyloid deposits (**B**). Structures resembling microglia are also found to be highlighted with the ADan immunohistochemical preparations in the pre-amyloid deposits (**C**, arrows). The bar represents 500 µm in (**A**) and 50 µm in (**B**) and 25 µm in (**C**). ADan immunohistochemistry (red, row **D** and **E**) combined with Thioflavin staining (green) highlights the presence of ADan in cells resembling microglia. Microglial antibodies were used to determine the Thioflavin positive structures identified in the cells (rows **F** and **G**, arrows). The bar represents 100 µm in row (**D**) and 20 µm in row (**E**) and 10 µm rows in (**F**) and (**G**). Data is from one donor

Based on the finding that *ITM2B/BRI2* expression is enriched in microglia, we sought to investigate the pathological contribution of microglia in mutant *ITM2B/BRI2*-associated post-mortem hippocampal tissue from one FBD case (Case 5 from Holton et al. 2001) and one FDD case (Case 3 from Holton et al. 2002). In situ hybridisation demonstrates that cells of both glial and neuronal morphology express *ITM2B*, albeit this technique is not quantitative (Fig S7) [31]. Staining was not undertaken on control brains as the focus of this study was to investigate the cellular location of the ABri and ADan which are absent in normal control brains. Both cases used in this study have undergone detailed pathological analysis which has been reported elsewhere [24, 25]. The hippocampus was selected based on previous knowledge of the underlying pathology in these cases. In both FBD and FDD, the hippocampus displays amyloid and pre-amyloid parenchymal deposits (Figs. 2A, 3A). Analyses employed antibodies against ABri (Ab338) and ADan (Ab5282) in conjunction with microglia markers and Thioflavin staining which highlights amyloid structures. In FBD, ABri was found in the form of amyloid and pre-amyloid plaques as previously documented [24] (Fig. 2A–C). The pre-amyloid diffuse ABri deposits contained microglia shaped cells stained positive by the ABri antibody (Fig. 2C, arrows). In regions of pre-amyloid deposition, fluorescent ABri immunohistochemistry (IHC) was undertaken with thioflavin staining to confirm that the cells contained ABri in an amyloid conformation (Fig. 2 row D and E, arrows). Double IHC confirmed that the amyloid was present within microglia using CD68 (Fig. 2 row F) and CR3/43 (Fig. 2 row G) microglial markers. We further explored ITM2B/BRI2-associated amyloid in a FDD case. ADan was found predominantly as pre-amyloid deposits in the hippocampus (Fig. 3A, B) and in the form of cerebral amyloid angiopathy. ADan immunohistochemistry clearly outlines microglia shaped cells (Fig. 3C, arrows). When visualised with Thioflavin and ADan, microglial morphologies were clearly visible surrounded by the pre-amyloid deposits (Fig. 3 rows D and E, arrows). Double IHC for microglial markers and Thioflavin, clearly showed the microglia containing Thioflavin positive amyloid (Fig. 3 rows F and G, arrows). Taken together, these findings suggest that ABri and ADan are found in an amyloidogenic conformation within the microglia when in close proximity to pre-amyloid deposits.

### Gene coexpression networks supports a role for ITM2B/BRI2 in disease associated microglial responses

**Fig. 4:**
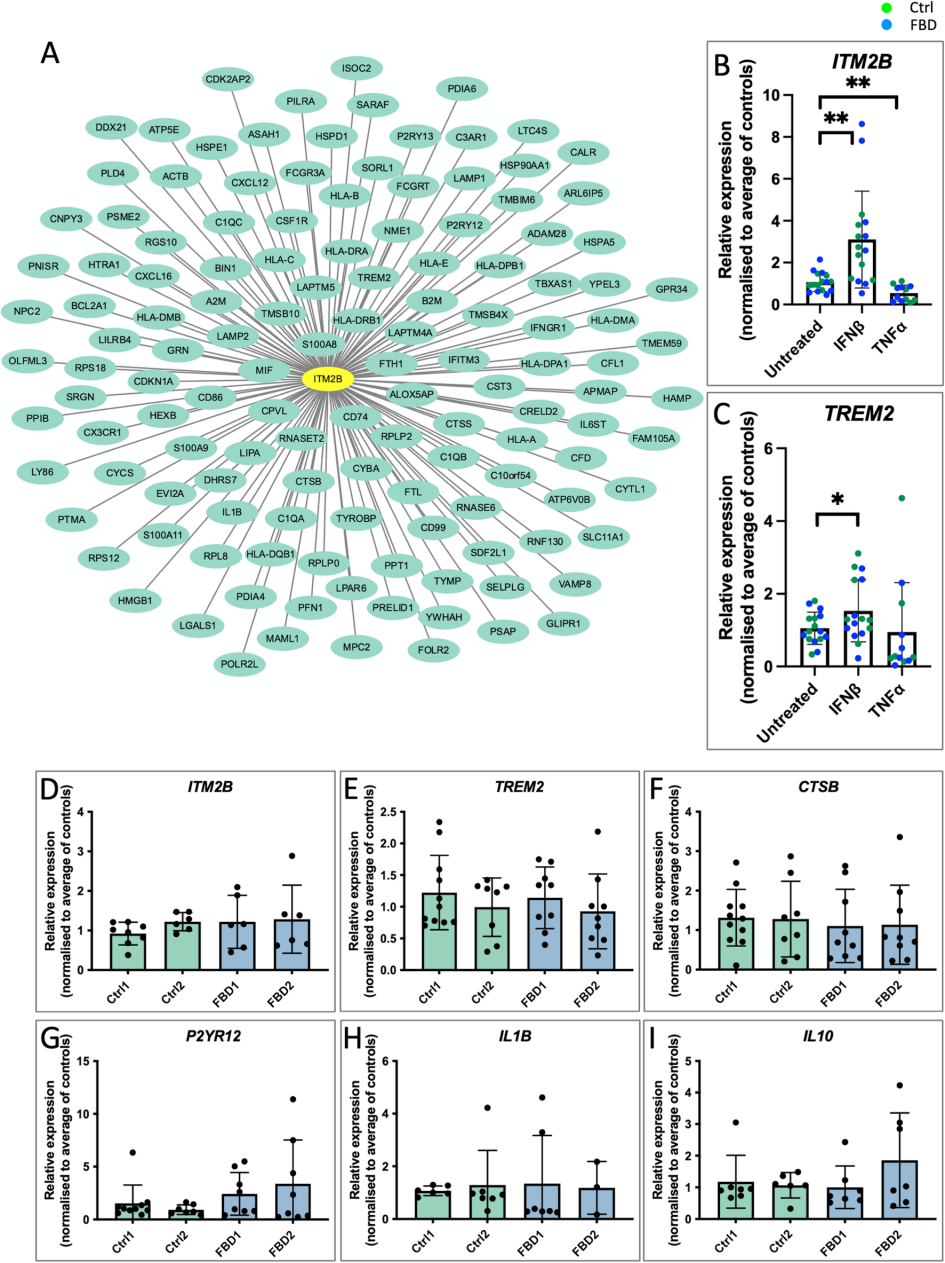
*ITM2B* is coexpressed with ARM network genes and responds to inflammatory cues in a similar manner to *TREM2*. **A** Genetic network plot of a module containing *ITM2B* detected in microglial cells isolated from human Alzheimer’s disease patients and individuals with MCI analysed by scRNA-seq [44] demonstrating *ITM2B* is coexpressed with ARM network genes in microglia isolated from human individuals showing neurodegeneration (and collectively varying between AD and MCI donors). Genes varying in response to AD with the highest connectivity to *ITM2B* from the co-expression network were plotted based on ranking the connectivity matrix of the expression data. This module contains genes associated with the DAM/ARM state (the full network and the strength of each interaction is given in Supplementary Table 1). Genes most strongly co-expressed with *ITM2B* include genes known to be associated with neurodegeneration including *LAPTM5*, HLA genes, *CTSB*, *CTSS*, *GRN*, *TREM2* and *TYROBP*. *ITM2B* is highlighted with a yellow oval. **B**, **C** qPCR expression analyses of *ITM2B* and *TREM2* in response to a 24 h treatment with IFNβ and TNFα. Data represent *n* = 4 for untreated and IFNβ and *n* = 3 for TNFα, each from 2 independent batches of microglia and for two control iPSC lines and two FBD lines. Data separated by genotype is presented in Fig S8B. Paired *t*-tests were performed for each treatment relative to untreated samples, * = *p* < 0.05, ** = *p* < 0.01. **D**–**I** qPCR analyses of genes associated with microglial state changes under basal conditions. Data represent 6 ≤ *n* ≤ 11 from 3 independent batches of microglia

Finally, to further explore the functional relevance of *ITM2B/BRI2* expression in microglial activation in neurodegeneration, we performed gene coexpression network analysis to reveal the gene networks and the microglial states in which *ITM2B/BRI2* was expressed. Using a single cell RNA-sequencing dataset of microglia isolated from human AD brain and individuals with mild cognitive impairment (MCI)[44], we performed an improved version of weighted gene coexpression network analysis (WGCNA[6]) to understand how networks or groups of genes collectively varied between AD and MCI. The network analysis revealed a high enrichment of ARM signature genes within the *ITM2B/BRI2* network in response to AD (Figs. 4, S8 and Supplementary Table 1). The genetic network containing *ITM2B/BRI2* contained known ARM genes such as *TREM2* and *TYROBP*, complement-associated genes (*C1QA*, *C1QB*), lysosome-related genes (*CTSB*, *CTSS*, *LAMP2*) and *HLA* genes. To reinforce these findings, we leveraged additional online databases to further investigate coexpression networks for *ITM2B/BRI2* (see methods). Genes displaying coexpression with *ITM2B/BRI2* included the HLA gene *B2M* and lysosome-related genes *LAMP2*, *GRN, LAMP1*, *LAPTM4A*, *PSAP* and *ASAH1* (Fig S9). Similar to *ITM2B/BRI2*, the genes *B2M*, *ASAH1*, *GRN*, and *PSAP* were among the top 100 most highly expressed genes in iPSC-derived microglia (Supplementary Table 2) [34] and are also enriched in ARM states in additional human and mouse datasets[27, 44, 53].

To further investigate the role of ITM2B in different microglial states, we treated control and patient-derived microglia with IFNβ and TNFα for 24 h and LPS for 6 h and then compared the response of *ITM2B* to the established ARM gene *TREM2* (Figs. 4B, C, S8B, C). IFNβ lead to an upregulation of both *ITM2B* and *TREM*, whereas TNFα and LPS lead to a downregulation of both genes. We did not observe a significant difference in the responses between control and *ITM2B* mutant microglia.

Finally, to investigate whether the FBD mutation or the presence of ABri in the media alters the inflammatory state of FBD microglia under basal conditions, we investigated the expression levels of ARM-associated genes (*ITM2B, TREM2* and *CTSB* Fig. 4D–F), the homeostatic gene *P2YR12* (Fig. 4G), as well as proinflammatory cytokine *IL1B* (Fig. 4H) and anti-inflammatory cytokine *IL10* (Fig. 4I). We observed no significant difference between control and FBD microglia.

Together, these data support a role for *ITM2B/BRI2* in the microglial response to damage and neurodegenerative diseases such as AD.

## Discussion

Here we describe a novel patient-derived iPSC model of FBD, providing a human physiological model of disease. Surprisingly, expression of *ITM2B/BRI2* was substantially higher in microglia compared with neurons and astrocytes. Consequently, we were able to detect ABri in patient-derived microglial cultures and not in the neuronal cultures used in this study, suggesting microglia represent a major source of ABri in FBD. This is an unexpected finding and contrasts with some existing literature [1], however, it is supported by recent work also demonstrating microglial enrichment of *Itm2b/Bri2* in mouse models [76].

ABri is a toxic peptide that has been shown to cause apoptotic cell death when administered to neuronal cell lines [9, 65]. Our finding supports the notion that in FBD, microglial-derived amyloidogenic peptides may contribute to plaque pathology. Given the pathological and clinical overlap between FBD and Alzheimer’s disease, it is intriguing to consider the amyloid cascade hypothesis [58] initiating via different amyloids from distinct cellular sources—converging on a disease pathogenesis featuring tau pathology, inflammation, neurodegeneration, and dementia-like symptoms.

Pathological examination of post-mortem tissue from FBD and FDD shows ABri/ADan colocalised with microglia in close proximity to pre-amyloid deposits. The presence of ABri and ADan in microglial cells in FBD and FDD, respectively, highlights a critical role for microglia in either amyloid production or uptake. Further investigations would be needed to determine the exact role microglia play in conversion of amyloidogenic peptides to amyloid. Published bulk expression data suggest that *ITM2B/BRI2* expression is highest in the hippocampus and the cerebellum (Fig. S10) [21, 51]. This is distinct from the expression pattern another microglial marker *TREM2*. The expression levels correlate with the occurrence of parenchymal pathology in FBD [24] and FDD [25], whereas CAA is found more widespread. The levels of pathology in these brain structures may help to explain the clinical symptoms of disease.

Our data, together with existing expression data from human and mouse cells, show that *ITM2B/BRI2* expression is enriched in microglia [11, 17, 22, 30, 36, 55, 77, 78]. Indeed, we searched data from Lin and colleagues who differentiated iPSC to neurons, astrocytes and microglia [34] and we saw that *ITM2B/BRI2* was within the top 100 highest expressed genes in the microglial lineage (based on normalised fragments per kilobase per million mapped fragments, Supplementary Table 2). Single cell data suggest that *ITM2B/BRI2* is enriched in ARM microglial clusters [44, 67]. This leads to two potential hypotheses: 1) induction of a DAM/ARM-like state induces expression of *ITM2B/BRI2*, which leads to the production and deposition of ABri and further disease progression. Alternatively, 2) a putative loss of function of ITM2B/BRI2 protein, as described in rodent models [64, 75], may negatively impact on the normal response of microglia to early pathological changes and cellular damage, thereby worsening the disease. We observed evidence for neither reduced ITM2B/BRI2 protein abundance in our FBD patient-derived microglial model nor altered inflammatory profiles of FBD microglia under basal conditions—supporting the first hypothesis or a combination of both. Indeed, the fact that IFNβ was able to upregulate *ITM2B* expression further supports this notion.

Clinically, minor accidents and trauma have been associated with symptom onset in FBD. For example, a flu-like disease was associated with disease onset in one of the patients from whom iPSCs were made in this study [20]. Whilst speculative, this might be compatible with a role for the immune system in FBD—whereby an inflammatory response to environmental factors may trigger or enhance expression of the pathological protein.

A caveat of using iPSC neurons is that they have a transcriptome largely resembling foetal neurons [45], which is a challenge for investigating genes whose expression changes throughout development. Thus, we cannot exclude that *ITM2B* expression in neurons may increase at extended time points as we have previously demonstrated for tau [62]. However, the cell type specific enrichment we observe in adult, human post-mortem tissue supports a predominantly microglial expression of *ITM2B/BRI2*. Nonetheless, this could be further investigated using protocols which promote the retention of signatures of neuronal maturity, such as transdifferentiation [39]. Despite the presence of ABri in iPSC-derived microglial cultures, we cannot discount the contribution of other cell types to ABri production; for example, endothelial cells and oligodendrocytes; especially given the high burden of angiopathy [24]. However, in situ hybridisation for *ITM2B/BRI2* in post-mortem tissue displayed weak signal in white matter and the vascular unit [31]. Neurons have been shown to express *ITM2B/BRI2* [1, 31, 57] and may upregulate ITM2B in a context dependent manner [8]. Although published studies focused on neurons and were limited to a single FBD case, our in-situ hybridisation studies reveal the presence of ITM2B/BRI2 expression in glial cells as well as neurons [31]. Context has been shown to control *ITM2B*/BRI2 expression in astrocytes [49]. Indeed, our iPSC-derived neuronal model does demonstrate low level expression, raising the possibility that immaturity of iPSC cultures may explain the low *ITM2B/BRI2* expression in these models. However, single cell sequencing datasets from adult human post-mortem tissue and from adult rodents (Figs. S3, S4) and a recently reported independent study [76] support the finding that microglia are a major contributing source of ITM2B/BRI2 and ABri. A limitation to this study is the fact that FBD is an extremely rare disease, meaning the number of patient-derived iPSC lines and access to brain tissue is limited. Future work will expand these data via the use of genome editing to increase the number of lines with which to investigate mechanisms of FBD as well as expand investigations to other *ITM2B*-linked dementias. Future work will also address the associated limitation that gene coexpression network analysis is done on Alzheimer’s disease brain datasets rather than tissue from FBD.

In summary, we propose a central role for microglial-derived ABri in FBD and subsequent non-cell autonomous mechanisms driving neuronal dysfunction. This surprising finding has relevance to the amyloid cascade hypothesis and Alzheimer’s disease; insomuch as 1) distinct origins of amyloidogenic peptides can culminate in neurodegeneration and dementia-like symptoms and 2) microglia and the immune response are central to disease onset as well as progression.

## Supplementary Information

Below is the link to the electronic supplementary material.

Supplementary file1 (PPTX 33282 KB)

Supplementary file2 (XLSX 17 KB)

Supplementary file3 (DOCX 20 KB)

## Data Availability

Cell lines and data generated within this study are available from the authors upon request.

## Abbreviations

AD: Alzheimer’s disease
ABri: Amyloid-Bri
APP: Amyloid precursor protein
ARM: Amyloid responsive microglia
CAA: Cerebral amyloid angiopathy
DAM: Disease-associated microglia
DMEM: Dulbecco’s modified eagle medium
FBD: Familial British dementia
MCI: Mild cognitive impairment
IHC: Immunohistochemistry
iPSC: Induced pluripotent stem cells
siRNA: Short interfering RNA
WGCNA: Weighted gene coexpression network analysis
