# Supplementary material for: Microglia contribute to the production of the amyloidogenic ABri peptide in familial British dementia
Source: Acta Neuropathol. 2024 Nov 15;148(1):65. doi: 10.1007/s00401-024-02820-z (PMC11568029; doi:10.1007/s00401-024-02820-z)
Supplement: Supplementary file 1 — Supplementary file1 (PPTX 33282 KB) [file 401_2024_2820_MOESM1_ESM.pptx]

## Slide 1
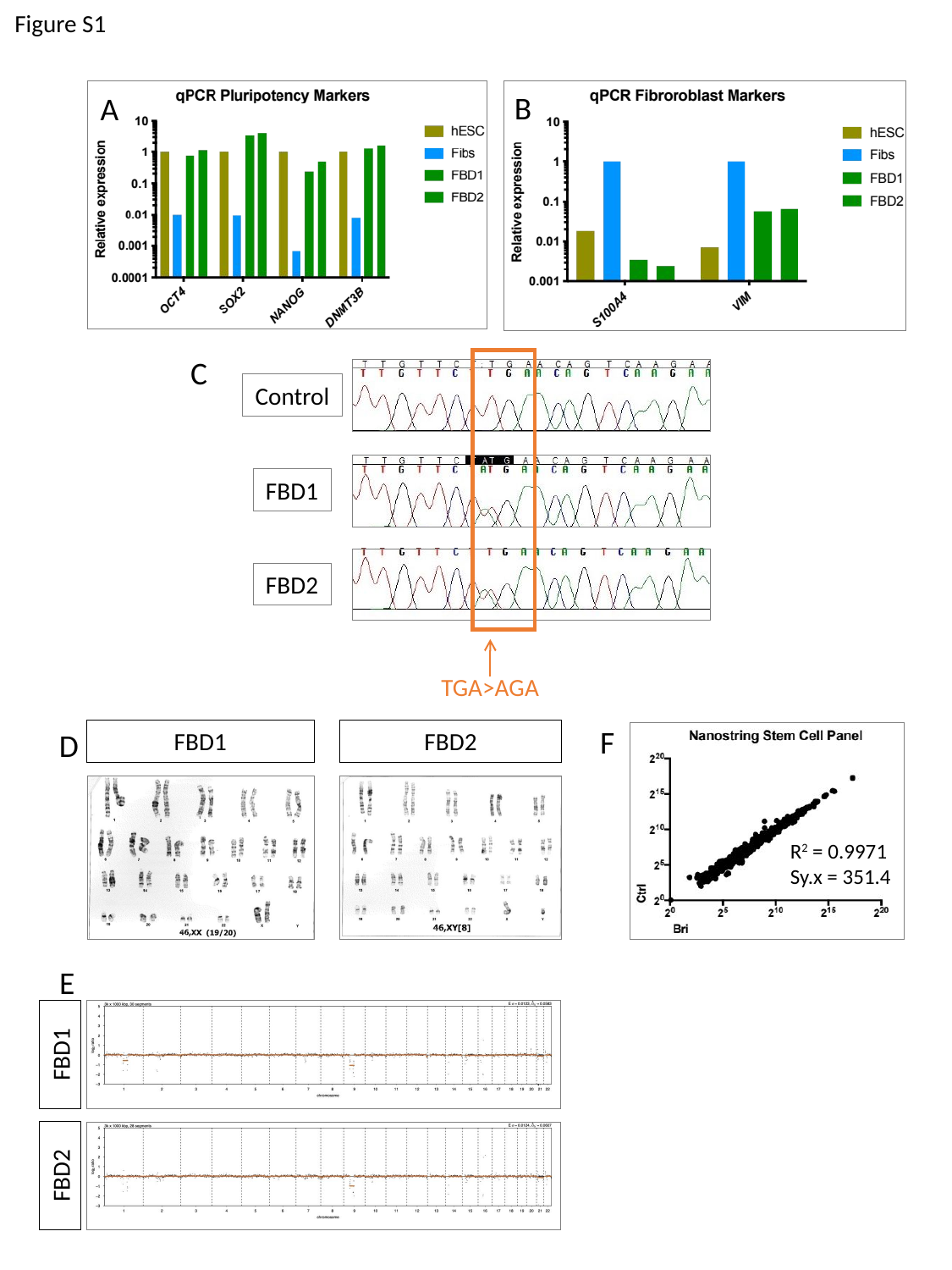

Figure S1
B
A
C
Control
FBD1
FBD2
TGA>AGA
F
D
FBD2
FBD1
R2 = 0.9971
Sy.x = 351.4
E
FBD1
FBD2

## Slide 2
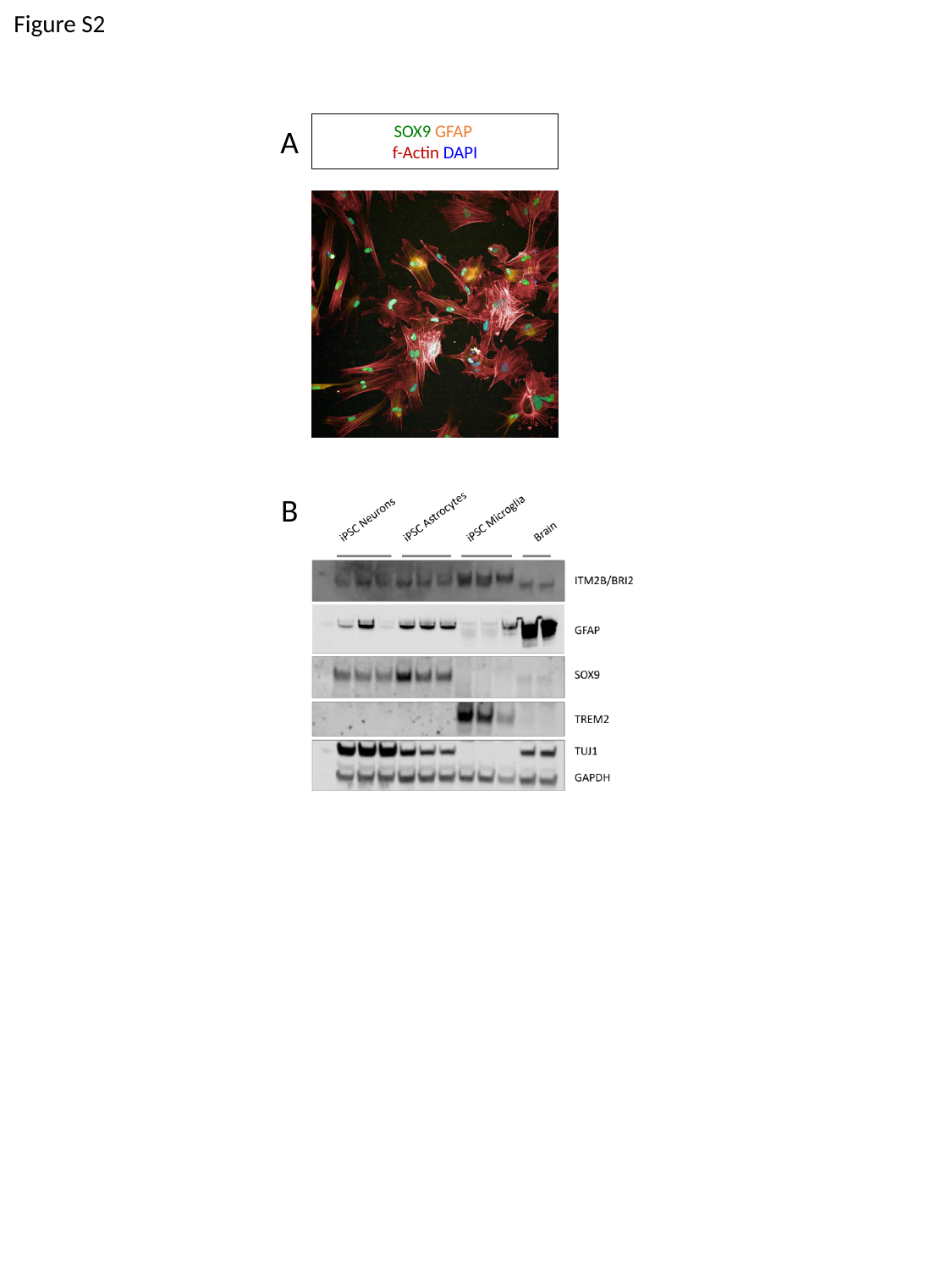

Figure S2
SOX9 GFAP
f-Actin DAPI
A
B

## Slide 3
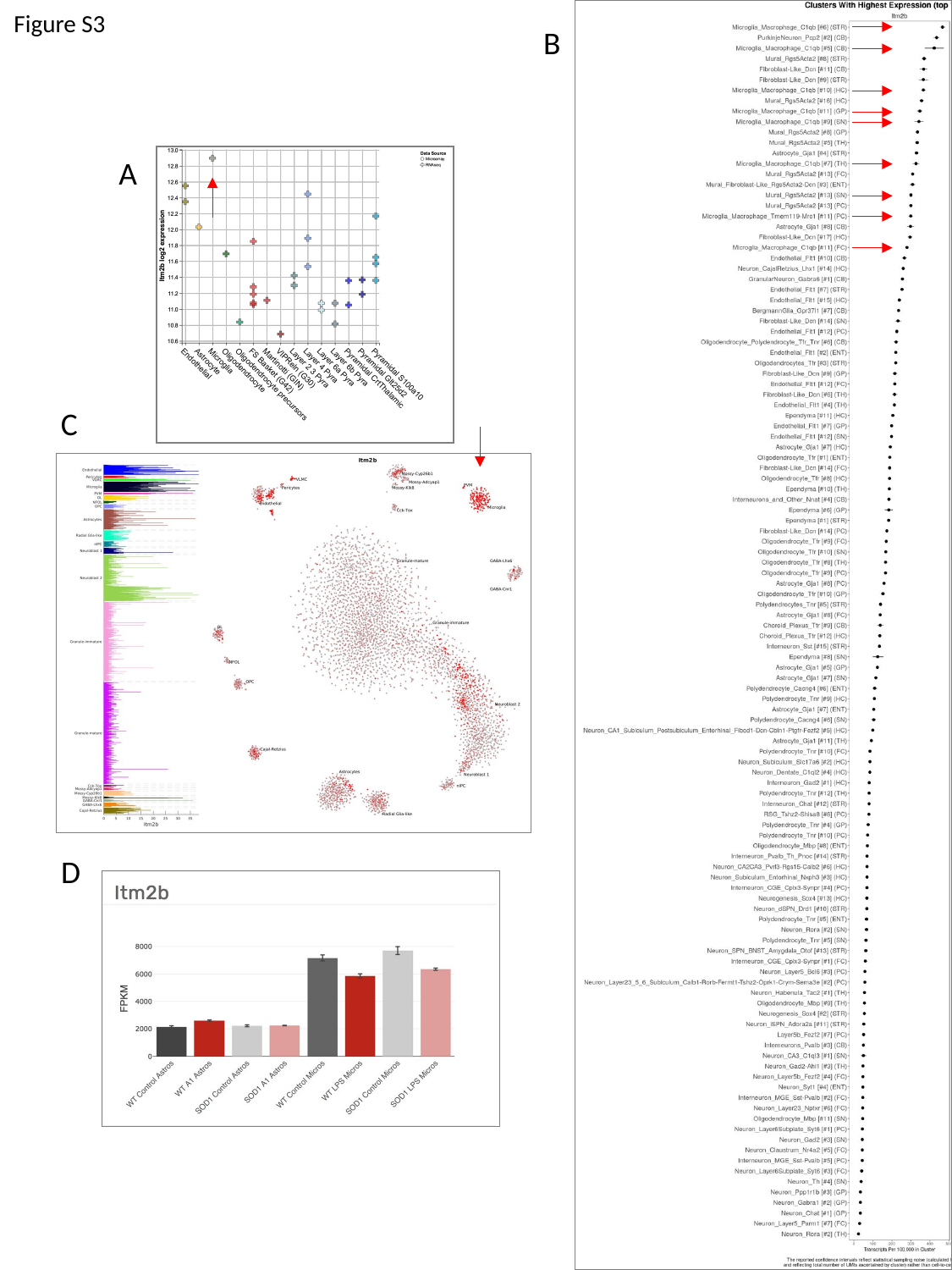

Figure S3
B
A
C
D

## Slide 4
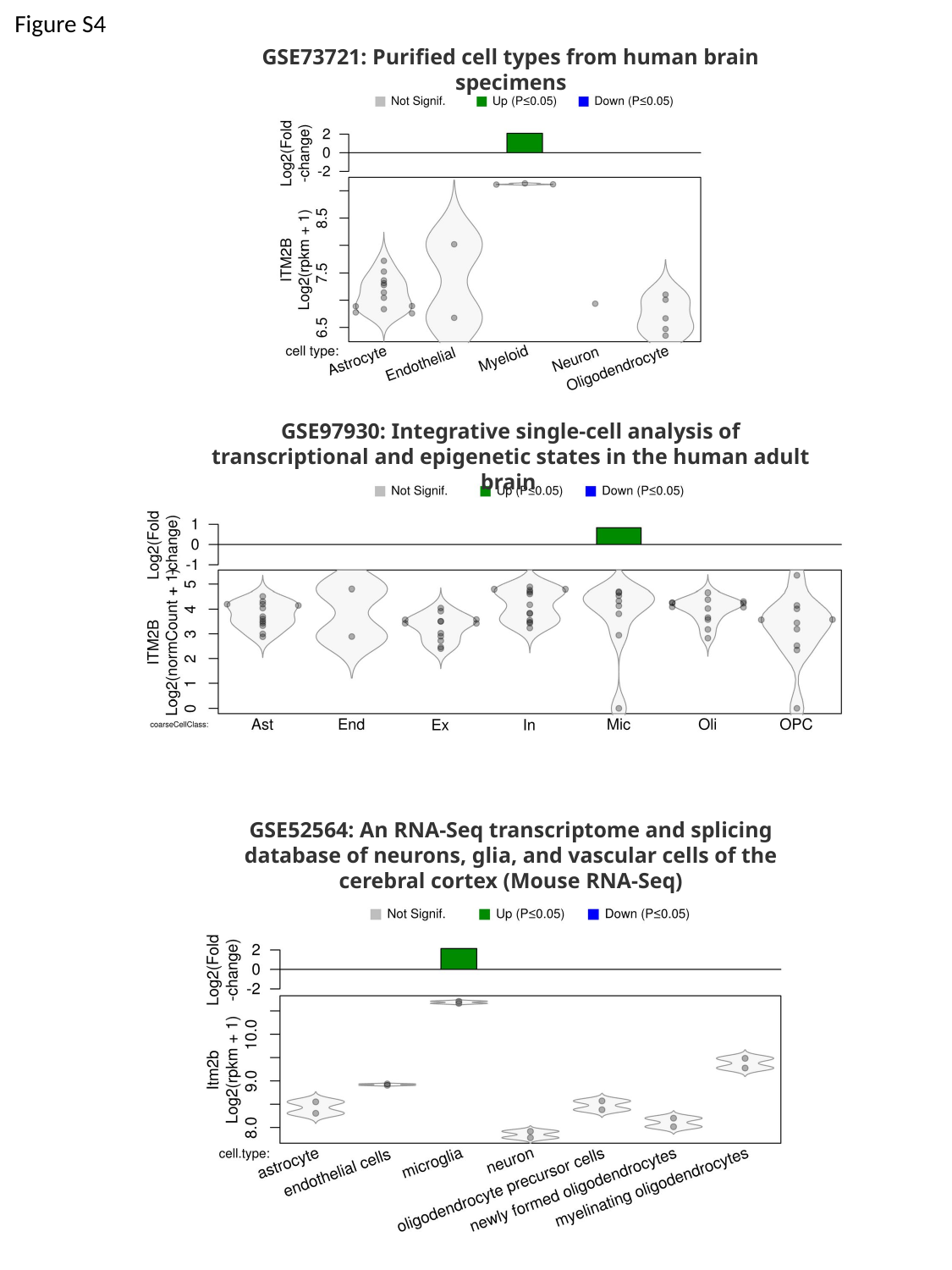

Figure S4
GSE73721: Purified cell types from human brain specimens
GSE97930: Integrative single-cell analysis of transcriptional and epigenetic states in the human adult brain
GSE52564: An RNA-Seq transcriptome and splicing database of neurons, glia, and vascular cells of the cerebral cortex (Mouse RNA-Seq)

## Slide 5
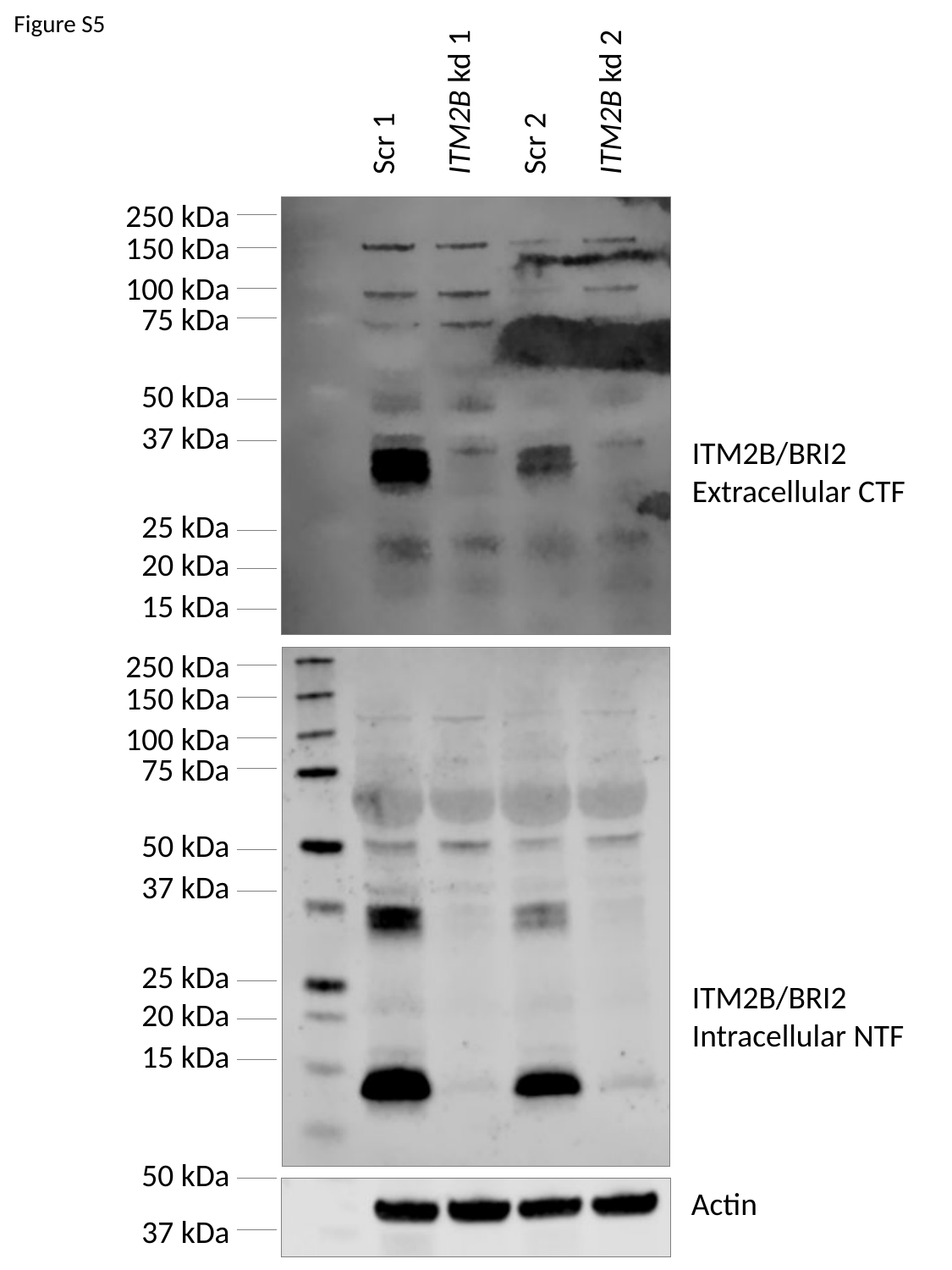

Figure S5
ITM2B kd 1
ITM2B kd 2
Scr 1
Scr 2
250 kDa
150 kDa
100 kDa
75 kDa
50 kDa
37 kDa
ITM2B/BRI2
Extracellular CTF
25 kDa
20 kDa
15 kDa
250 kDa
150 kDa
100 kDa
75 kDa
50 kDa
37 kDa
25 kDa
ITM2B/BRI2
Intracellular NTF
20 kDa
15 kDa
50 kDa
Actin
37 kDa

## Slide 6
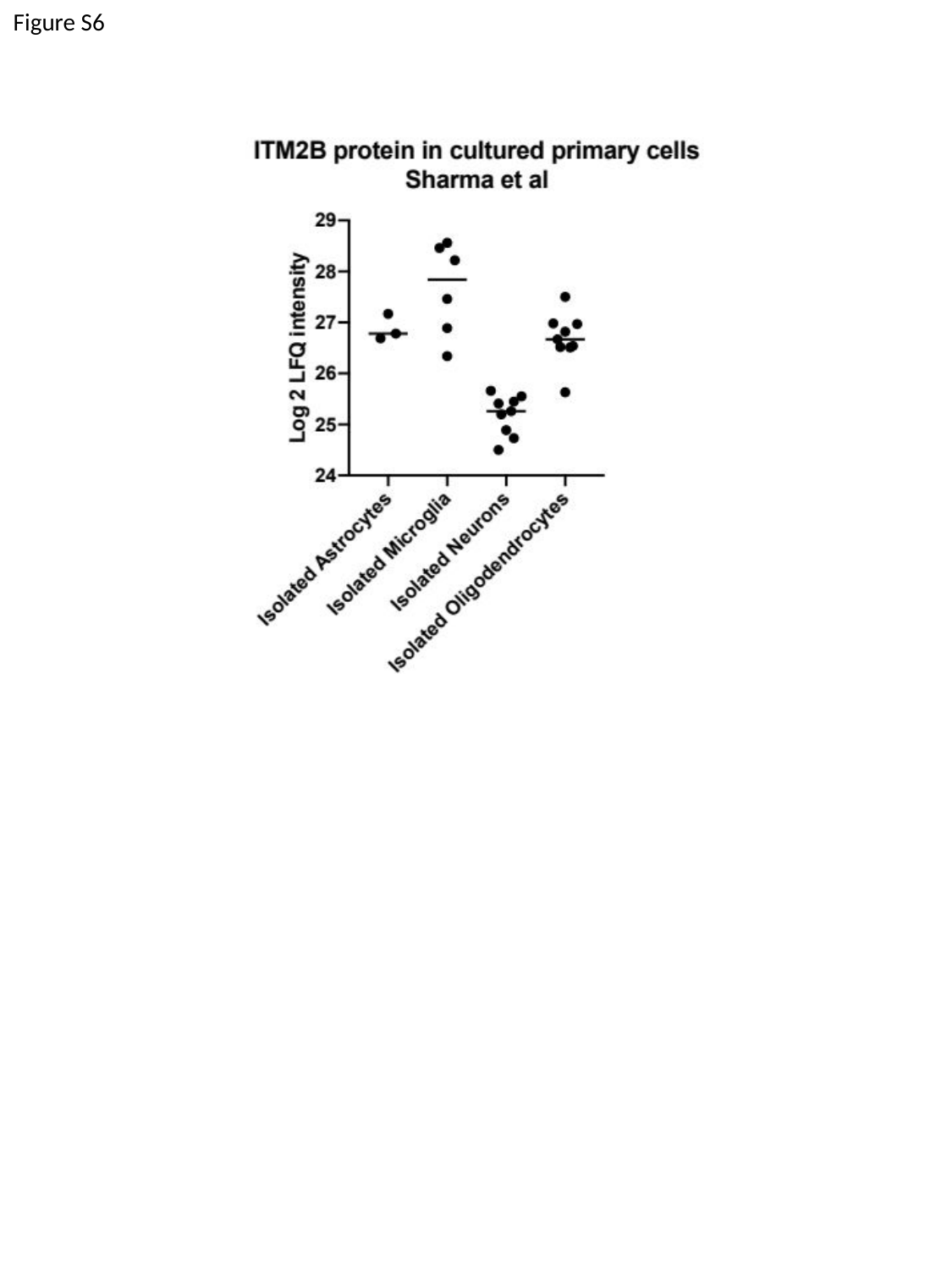

Figure S6

## Slide 7
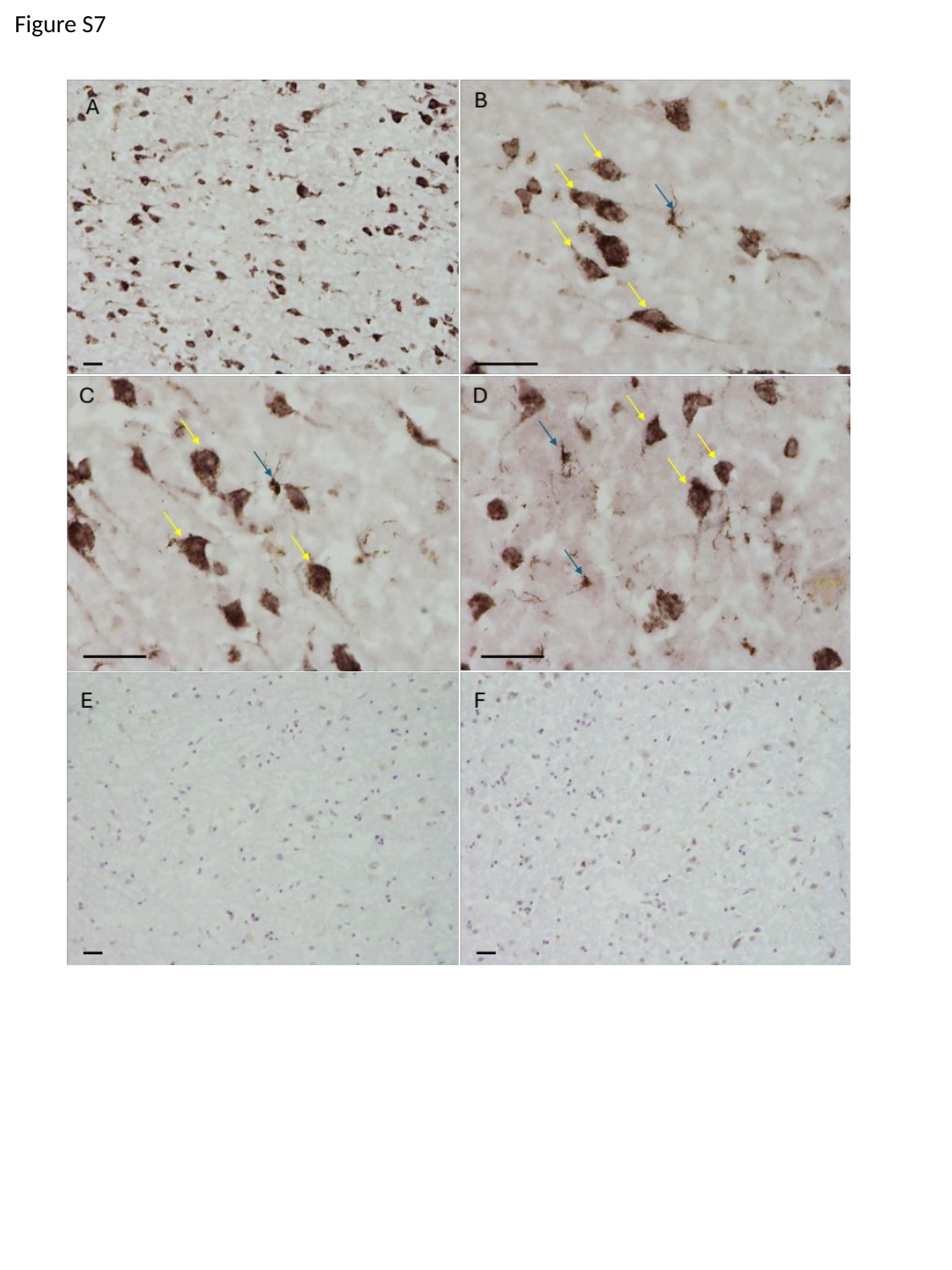

Figure S7

## Slide 8
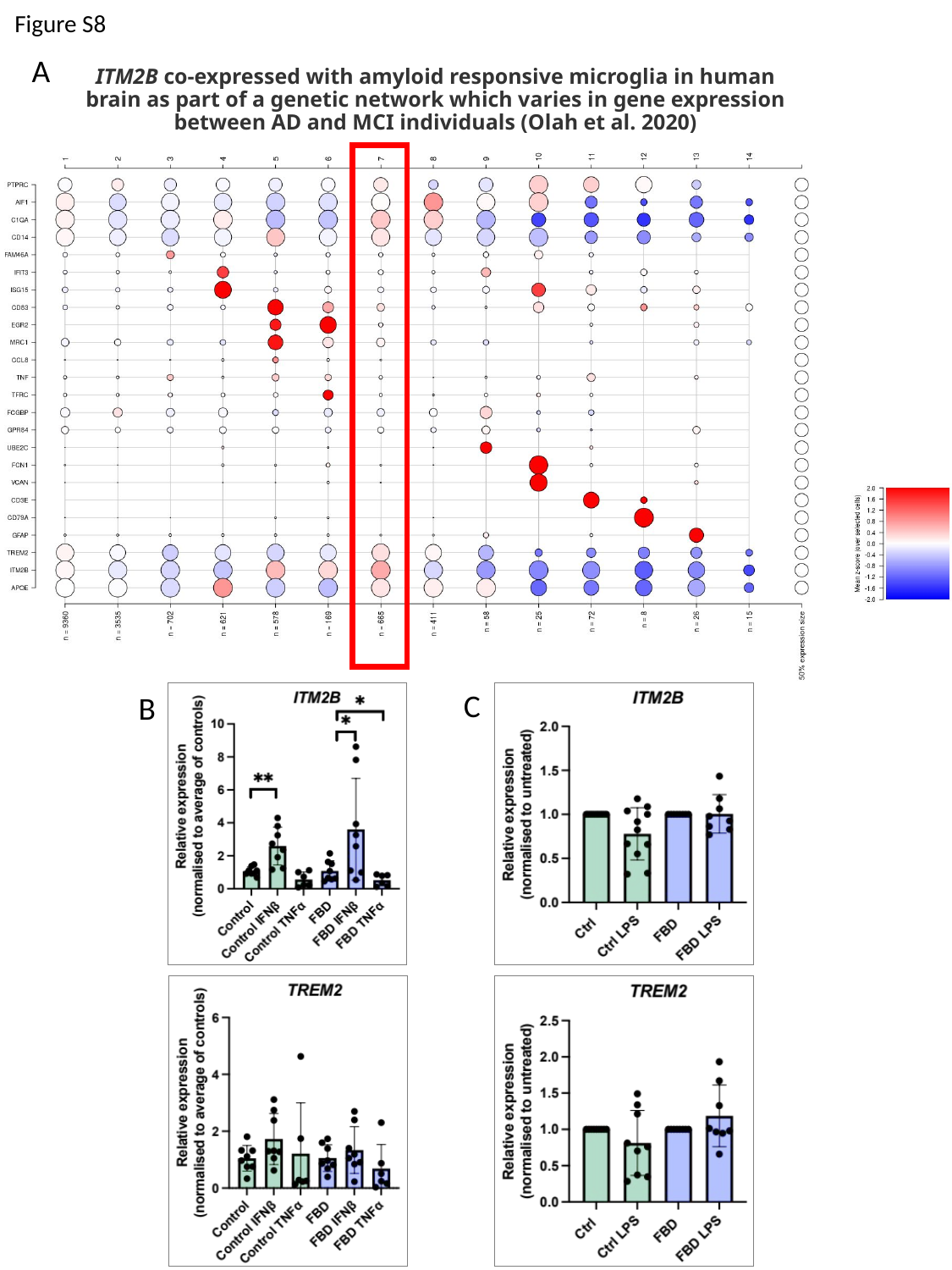

Figure S8
A
ITM2B co-expressed with amyloid responsive microglia in human brain as part of a genetic network which varies in gene expression between AD and MCI individuals (Olah et al. 2020)
C
B

## Slide 9
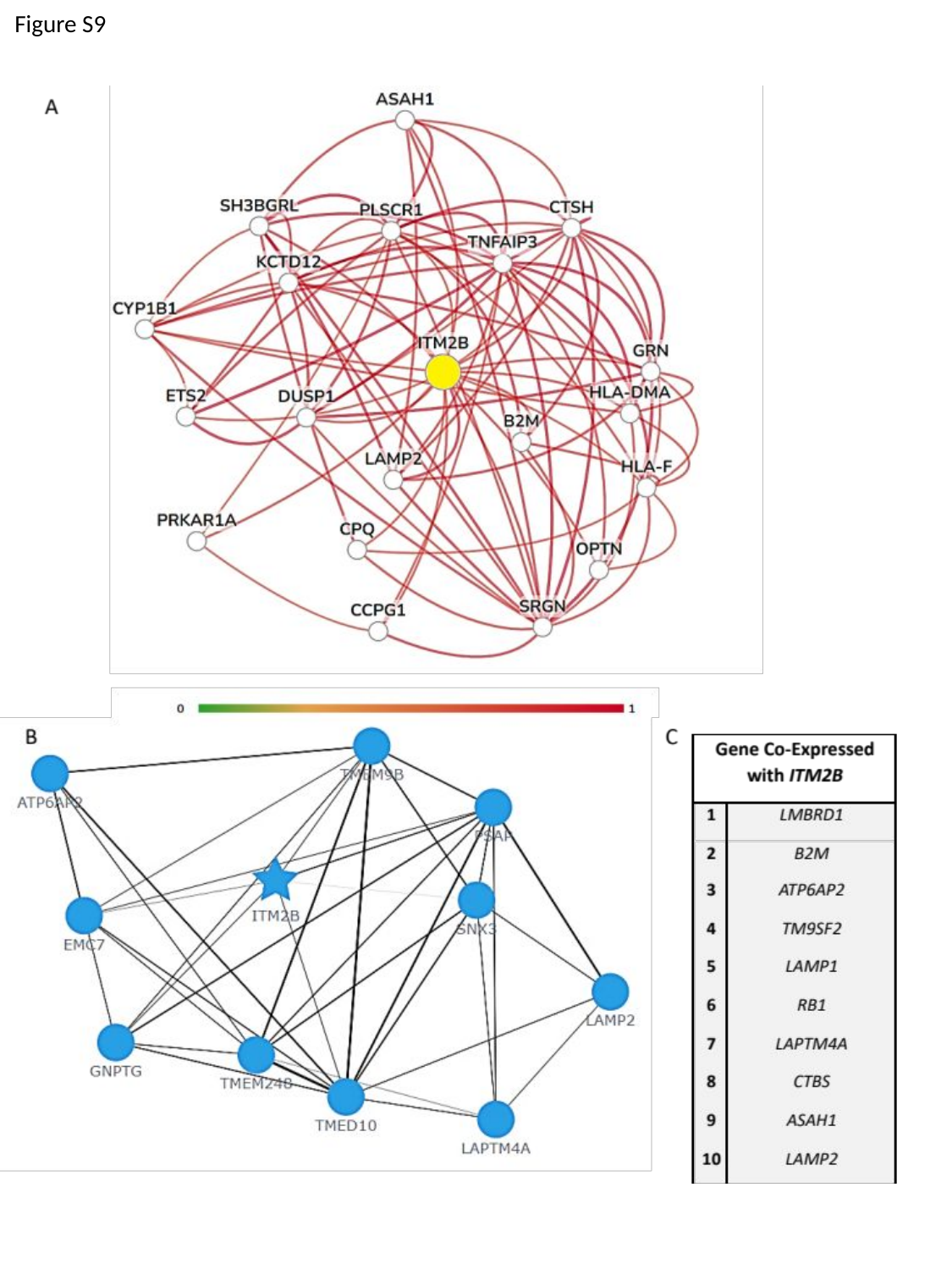

Figure S9

## Slide 10
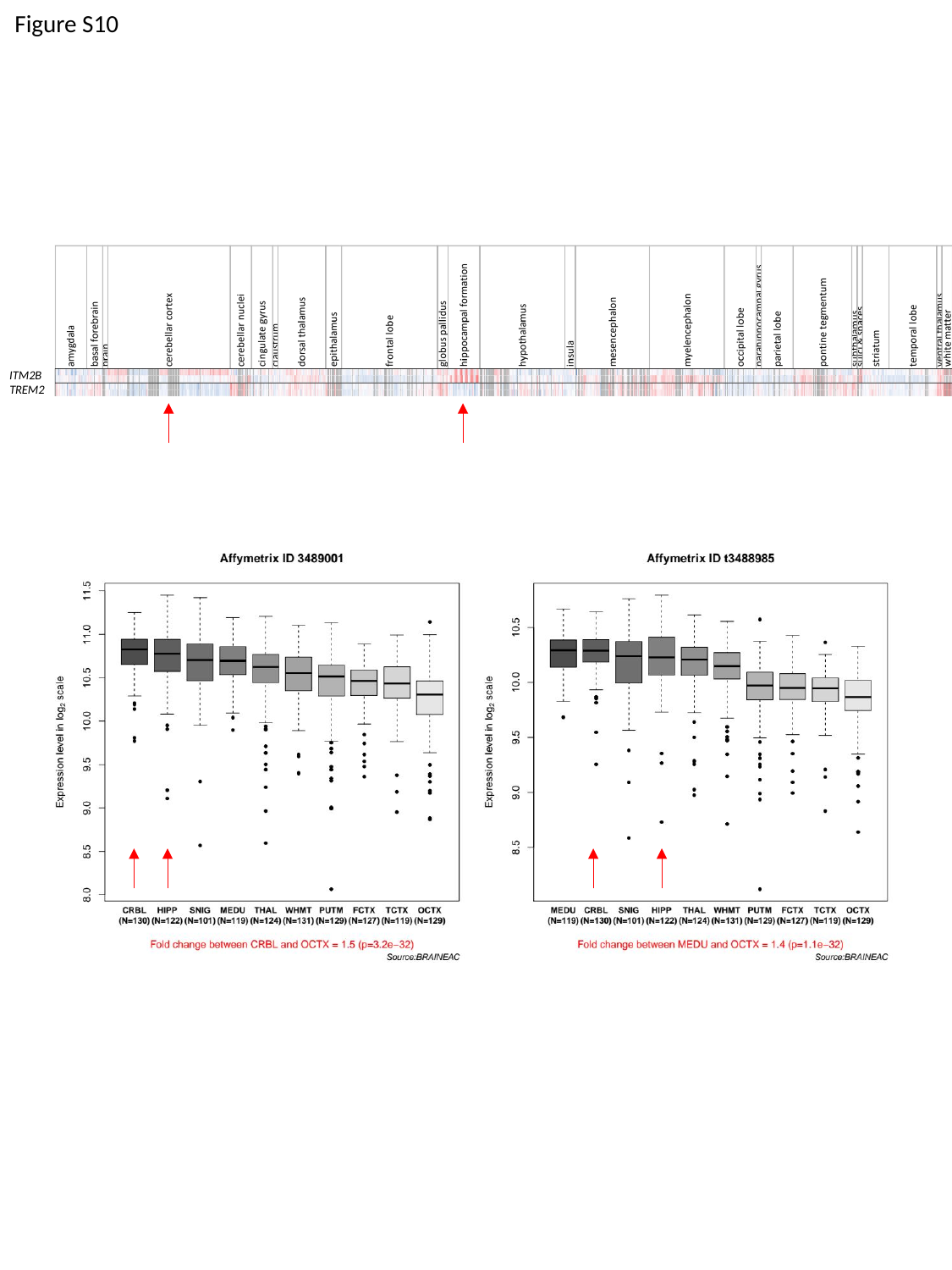

Figure S10
ITM2B
TREM2
