# Supplementary material for: Microglia contribute to the production of the amyloidogenic ABri peptide in familial British dementia
Source: Acta Neuropathol. 2024 Nov 15;148(1):65. doi: 10.1007/s00401-024-02820-z (PMC11568029; doi:10.1007/s00401-024-02820-z)
Supplement: Supplementary file 3 — Supplementary file3 (DOCX 20 KB) [file 401_2024_2820_MOESM3_ESM.docx]

| **1** | *FTL* |
| --- | --- |
| **2** | *TMSB4X* |
| **3** | *FTH1* |
| **4** | *ACTB* |
| **5** | *SPP1* |
| **6** | *PSAP* |
| **7** | *TPT1* |
| **8** | *CTSD* |
| **9** | *B2M* |
| **10** | *ACTG1* |
| **11** | *CTSB* |
| **12** | *CTSZ* |
| **13** | *RPS27* |
| **14** | *COL3A1* |
| **15** | *RPL37A* |
| **16** | *GPNMB* |
| **17** | *COL1A1* |
| **18** | *LGALS1* |
| **19** | *LGMN* |
| **20** | *TMSB10* |
| **21** | *CD68* |
| **22** | *RPS29* |
| **23** | *GPX1* |
| **24** | *RPL23* |
| **25** | *S100A11* |
| **26** | *SEPP1* |
| **27** | *RPLP1* |
| **28** | *RPL13AP5* |
| **29** | *C1QC* |
| **30** | *RPL38* |
| **31** | *RPS6* |
| **32** | *CD63* |
| **33** | *RPL31* |
| **34** | *RPS12* |
| **35** | *MYL6* |
| **36** | *RPS4X* |
| **37** | *RPLP2* |
| **38** | *PFN1* |
| **39** | *CFL1* |
| **40** | *FCER1G* |
| **41** | *RPL27* |
| **42** | *RPL30* |
| **43** | *NPC2* |
| **44** | *RPL19* |
| **45** | *RPS11* |
| **46** | *TYROBP* |
| **47** | *ATP5E* |
| **48** | *RPS18* |
| **49** | *COL1A2* |
| **50** | *SPARC* |
| **51** | *VIM* |
| **52** | *RPS28* |
| **53** | *RPL35* |
| **54** | *CSTB* |
| **55** | *RPS24* |
| **56** | *RPS16* |
| **57** | *LAPTM5* |
| **58** | *RPL11* |
| **59** | *C1QB* |
| **60** | *EEF1A1* |
| **61** | *IFI30* |
| **62** | *GAPDH* |
| **63** | *DLK1* |
| **64** | *RPS15A* |
| **65** | *GRN* |
| **66** | *TUBA1B* |
| **67** | *RPL35A* |
| **68** | *H19* |
| **69** | *RPS8* |
| **70** | *CD14* |
| **71** | *LIPA* |
| **72** | *RPS20* |
| **73** | *ANXA2* |
| **74** | *CD81* |
| **75** | *RPL26* |
| **76** | *ITM2B* |
| **77** | *CST3* |
| **78** | *RPS2* |
| **79** | *SOD2* |
| **80** | *FUCA1* |
| **81** | *RPL7A* |
| **82** | *GNB2L1* |
| **83** | *SAT1* |
| **84** | *OAZ1* |
| **85** | *RPL3* |
| **86** | *ATP6AP2* |
| **87** | *EEF2* |
| **88** | *RPLP0* |
| **89** | *RPS14* |
| **90** | *F13A1* |
| **91** | *ARHGDIB* |
| **92** | *CAPG* |
| **93** | *ATP6V0C* |
| **94** | *GNAS* |
| **95** | *FAU* |
| **96** | *RPL8* |
| **97** | *ASAH1* |
| **98** | *C1QA* |
| **99** | *CTSH* |
| **100** | *SDCBP* |

Supplementary Table 2
